# News Waves: Hard News, Soft News, Fake News, Rumors, News Wavetrains

**DOI:** 10.3390/e26010005

**Published:** 2023-12-19

**Authors:** Nikolay K. Vitanov, Zlatinka I. Dimitrova, Kaloyan N. Vitanov

**Affiliations:** 1 Institute of Mechanics, Bulgarian Academy of Sciences, Acad. G. Bonchev Str., Bl. 4, 1113 Sofia, Bulgaria; zdim@imbm.bas.bg (Z.I.D.); kalovitanov@gmail.com (K.N.V.); 2 Climate, Atmosphere and Water Research Institute, Bulgarian Academy of Sciences, Blvd. Tzarigradsko Chaussee 66, 1784 Sofia, Bulgaria

**Keywords:** nonlinear differential equations, simple equations method, exact solutions, SIR model of epidemics spread, news waves, time horizon, news wavetrain, effective reproduction number, hard news, soft news

## Abstract

We discuss the spread of a piece of news in a population. This is modeled by SIR model of epidemic spread. The model can be reduced to a nonlinear differential equation for the number of people affected by the news of interest. The differential equation has an exponential nonlinearity and it can be approximated by a sequence of nonlinear differential equations with polynomial nonlinearities. Exact solutions to these equations can be obtained by the Simple Equations Method (SEsM). Some of these exact solutions can be used to model a class of waves associated with the spread of the news in a population. The presence of exact solutions allow to study in detail the dependence of the amplitude and the time horizon of the news waves on the wave parameters, such as the size of the population, initial number of spreaders of the piece of the news, transmission rate, and recovery rate. This allows for recommendations about the change of wave parameters in order to achieve a large amplitude or appropriate time horizon of the news wave. We discuss five types of news waves on the basis of the values of the transmission rate and recovery rate—types A, B, C, D, and E of news waves. In addition, we discuss the possibility of building wavetrains by news waves. There are three possible kinds of wavetrains with respect of the amplitude of the wave: increasing wavetrain, decreasing wavetrain, and mixed wavetrain. The increasing wavetrain is especially interesting, as it is connected to an increasing amplitude of the news wave with respect to the amplitude of the previous wave of the wavetrain. It can find applications in advertising, propaganda, etc.

## 1. Introduction

The spread of news in a population is an important research problem [1,2,3] with large significance for the practice. Examples are conspiracy theories [4,5,6,7], echo chambers [8,9,10,11,12,13], formation of mass opinion [14], prejudice [15], network propaganda [16], exposure to ideologically diverse news [17], etc. [18,19]. Special attention is given to the spread of misinformation and fake news [20,21,22,23,24,25,26,27,28,29,30,31,32,33]. Let us also note the spread and misinformation and fake news in the time of the COVID-19 pandemics [34,35,36,37,38,39,40,41].

Below, we will use results about the exact solutions connected to the SIR model of epidemics in order to perform an analytical study of waves of news. There exist various models for spreading of an epidemic in a population and a large amount of literature is devoted to this (for several examples see [42,43,44,45,46,47,48,49,50,51,52,53,54]). Epidemic models can also be applied for description of other processes, such as the spreading of ideas, for example (for an overview see [55]).

The text is organ ized as follows. In Section 2, we briefly discuss the use of the SIR model of epidemics spread to model the spread of a piece of news in a population as well as the reduction of the model equations to a chain of nonlinear differential equations. We present several exact solutions to the equations of this chain. The solutions are obtained by the Simple Equations Method (SEsM). The method is briefly described in Appendix A and the process of obtaining the solutions is illustrated in Appendix B. In Section 3, we use some of the obtained solutions to derive analytical relationships for the three populations participating in the SIR model. In Section 4, we connect the obtained results to the spread of the news. Section 5 is devoted to a discussion of the possibilities for manipulation of the amplitude and the time horizon of the news wave. We distinguish five types of news waves with respect to the values of the transmission rate and recovery rate of the population where the wave spreads. Several concluding remarks are summarized in Section 6.

## 2. The SIR Model of Epidemics as a Model for Spread of News

The SIR model of epidemic spread can be reduced to a single nonlinear differential equation [56,57]. Then, the obtained equation can be associated with a chain of nonlinear differential equations which contain polynomial nonlinearities. We will use this approach to obtain analytical solutions which can be connected to the spread of news waves.

The model of news waves is obtained as follows [58]. Let us consider a population of *N* individuals. The population is divided into three groups with respect to some piece of news. There is a subpopulation of the potential authors of spread of the piece of news—*S*. Then, there is a subpopulation of authors, active in posting piece of news—*I*. Finally, there is a subpopulation of authors, which became inactive in spreading of the piece of news after some period of activity in spreading that piece of news—*R*. The model equations for the time change of the numbers of individuals from the above three subpopulations are as follows:

(1) $$\begin{array}{ccc} \frac{dS}{dt} & = & -\frac{\tau }{N}SI\\ \frac{dI}{dt} & = & \frac{\tau }{N}SI-\rho I\\ \frac{dR}{dt} & = & \rho I. \end{array}$$

where τ is the transmission rate (quantitative characteristics of the transition from the subpopulation of the potential spreaders to the subpopulation of active spreaders) and ρ is the recovery rate (quantitative characteristics of the transition from the subpopulation of active spreaders to the population of individuals who are not interested in the spread of the piece of the news). We consider below the most simple case, where these rates are assumed to be constants. We are going to discuss analytical results for this simple case. These analytical results can serve as orientation for the numerical study of more complicated cases.

From (1), we obtain the relationship N=S+I+R. *N* is the total population, which is assumed to be constant. In several more words, we assume that the changes in the total population are negligible for the time of the studied phenomenon (the news wave). We also stress the following. The system (1) is written for the spreaders of information. We can write the same system for people who hear some piece of news (hard news, soft news, fake news, etc.). In this case, *S* will be the subpopulation of individuals who are susceptible to the news (who can hear the corresponding piece of news). *I* will be the subpopulation of individuals who have heard the piece of the news and are interested in spreading that piece of news. Finally, *R* will be the subpopulation of individuals who are not interested anymore in the corresponding piece of news. In such a way, the model (1) allows us to study the process of spreading of the piece of news among the population of potentially interested people. The news can be classified according to different characteristics. They can be hard news or soft news, true news, or fake news. What will be of interest to us are the values of the transmission rate τ and the recovery rate ρ.

Below, we will obtain analytical relationships for R(t). On the basis of these relationships, we can calculate the number I(t) from the SIR model. This happens on the basis of the last equation of (1):

(2) $$I=\frac{1}{\rho }\frac{dR}{dt}.$$

On the basis of I(t), we calculate the growth rate:

(3) $$\sigma \left(t\right)=\frac{1}{I}\frac{dI}{dt}.$$

The growth rate σ(t) can be written as $\sigma \left(t\right)=\rho \left(R_{n}-1\right)$. Then:

(4) $$R_{n}\left(t\right)=1+\frac{\sigma (t)}{\rho }.$$

where $R_{n}\left(t\right)$ is called the time varying effective reproduction number for the spread of the news wave. There exists a specific value: $R_{n}=1$. If $R_{n}<1$, then σ(t)<0 and the relative growth rate is negative. This means that dI/dt is negative. In other words, the number of people, who post the piece of news (respectively, the number of people who have heard the news and are interested in that piece of news) will decrease and the significance of the corresponding piece of news will decrease. If $R_{n}>1$, then σ(t)>0 and the relative growth rate is positive. This means that dI/dt is positive. In other words, the number of the people who post the piece of news (respectively, the number of people who have heard the news and are interested in it) increases and the significance of the corresponding piece of news increases.

Another parameter of the news wave is its maximum $I_{m}$. This maximum is achieved for some time $t_{m}$ after the initial moment. $t_{m}$ is called the time horizon of the news wave. The time horizon of the news wave is an important characteristic and it will be mentioned many times below in the text.

Finally, the substitution of (2) in the first equation of (1) leads to:

(5) $$S=S\left(0\right)\exp\left\{-\frac{\tau }{\rho N}\left[R-R\left(0\right)\right]\right\}.$$

where S(0) and R(0) are the corresponding quantities at the time t=0.

What remains is to obtain an equation for R(t). The substitution of N=S+I+R and (5) in the last equation of (1) leads to the differential equation for *R*:

(6) $$\frac{dR}{dt}=\rho \left\{N-R-S\left(0\right)\exp\left[-\frac{\tau }{\rho N}\left(R-R\left(0\right)\right)\right]\right\}$$

We assume R(0)=0—no inactive news spreaders (respectively, no individuals, which are uninterested in the piece of the news) at t=0. We note that the case R(0)≠0 can be easily incorporated in the theory, presented below. One has just to substitute S(0) by $S{\left(0\right)}^{*}=S\left(0\right)\exp\left[\frac{\tau R(0)}{\rho N}\right]$.

Several results exist for the analytical solution to the equations of the SIR model. We mention the result connected to an integro-differential equation [59] as well as the implicit solution based on the Lambert function [60]. Below, we follow an idea which will lead us to an explicit exact analytical solution to (6). The solution will be for a specific class of situations where the ratio $\frac{\tau R}{\rho N}$ is small enough. This can be realized, for an example when *R* is small enough in comparison to *N*. Note that *N* is the total population. Let, for example, N=10,000,000. Let us consider a wave which maximum value is $R_{max}=100$,000. τ and ρ usually are of the same order and for this case the maximum value of $x=\frac{\tau R}{\rho N}$ will be x=0.01. This value is small and exp(x) has maximum value of 1.010050167. Thus, there is no problem to expand exp(x) or exp(−x) in Taylor series despite the fact that *R* can have large values.

In other words, the main assumption is that the news wave affects a relatively small number of the individuals of the population. For the case of such waves, $\exp\left[-\frac{\tau }{\rho N}R\right]$ can be represented as a Taylor series $\exp\left[-\frac{\tau }{\rho N}R\right]=\sum_{j=0}^{M}\frac{1}{j!}{\left(-\frac{\tau }{\rho N}R\right)}^{j}$. The assumption here is that $\frac{e}{(M+1)!}{\left(\frac{\tau R}{\rho N}\right)}^{M+1}<<1$.

*M* has infinite value in the full Taylor series. However, we can truncate it at M=2, M=3,…, if $-\frac{\tau }{\rho N}R$ is small enough. From (6), we obtain:

(7) $$\frac{dR}{dt}=\rho \left\{N-R-S\left(0\right)\sum_{j=0}^{M}\frac{1}{j!}{\left(-\frac{\tau }{\rho N}R\right)}^{j}\right\},\;\;\;M=2,3,\cdots$$

We assume:

(8) $$\alpha_{0}=\rho \left[N-S\left(0\right)\right];\;\;\;\alpha_{1}=\frac{\tau S(0)}{N}-\rho ;\;\;\;\alpha_{j}=-\frac{{\left(-1\right)}^{j}}{j!}\frac{\tau^{j}S\left(0\right)}{\rho^{j-1}N^{j}},\;\;j=2,3,\cdots$$

Moreover, (7) becomes

(9) $$\frac{dR}{dt}=\sum_{j=0}^{M}\alpha_{j}R^{j}$$

We assume that ρ and τ are positive. Then, $a_{0}$ will have a positive value. The value of $a_{1}$ can be positive or negative. The values of $a_{j}$ is negative for even *j* and positive for odd *j*. We note that a similar reduction to a chain of equations can also be made for the SEIR model of epidemic spread [61].

The sets of Equations (7) and (9) are connected to the orders of approximation of (6). We can obtain exact solutions of these equations on the basis of the Simple Equations Method (SEsM) (see the Appendix A). The solutions which will be discussed here are listed below. The detail about obtaining these solutions are given in Appendix B.

We will discuss the following solutions of Equation (9). For the case M=2, we discuss the solution:

(10) $$R\left(t\right)=-\frac{\alpha_{1}}{2\alpha_{2}}-\frac{\theta }{2\alpha_{2}}\tanh\left[\frac{\theta (t+C)}{2}\right]+\frac{D}{\cosh^{2}\left[\frac{\theta (t+C)}{2}\right]\left\{E-\frac{2\alpha_{2}D}{\theta }\tanh\left[\frac{\theta (t+C)}{2}\right]\right\}}.$$

We can write G=D/E and this will reduce the number of the constants of integration from 3 to 2 for the case E≠0. In order to keep the case E=0 as a possibility, (10) is in the form without introduction of *G*. We note that when D=0, one obtains the specific solution known since [56].

For the cases M>2, we will discuss below solutions obtained for L=1. For the meaning of *L* see (A7) from Appendix B. For the case M=3, L=1 we discuss the solution:

(11) $$\begin{array}{c} R\left(t\right)=-\frac{\alpha_{2}}{3\alpha_{3}}+\beta_{1}{\left\{\frac{3\alpha_{1}\alpha_{3}-\alpha_{2}^{2}}{-3\alpha_{3}^{2}\beta_{1}^{2}+C\left(3\alpha_{1}\alpha_{3}-\alpha_{2}^{2}\right)\exp\left\{-2\frac{3\alpha_{1}\alpha_{3}-\alpha_{2}^{2}}{3\alpha_{3}}t\right\}}\right\}}^{\frac{1}{2}} \end{array}$$

We can write additional solutions for larger values of *M*. For example, let us consider the case M=4, L=1. The solution is:

(12) $$\begin{array}{c} R\left(t\right)=-\frac{\alpha_{3}}{4\alpha_{4}}+\beta_{1}{\left\{\frac{\alpha_{3}^{4}-256\alpha_{0}\alpha_{4}^{3}}{64\beta_{1}^{3}\alpha_{3}\alpha_{4}^{3}+C\left(\alpha_{3}^{4}-256\alpha_{0}\alpha_{4}^{3}\right)\exp\left\{3\frac{\alpha_{3}^{4}-256\alpha_{0}\alpha_{4}^{3}}{64\alpha_{3}\alpha_{4}^{2}}t\right\}}\right\}}^{\frac{1}{3}}. \end{array}$$

Next, we consider the case M=5, L=1. The solution is:

(13) $$\begin{array}{c} R=-\frac{\alpha_{4}}{5\alpha_{5}}+\beta_{1}{\left\{\frac{-\alpha_{4}^{5}+3125\alpha_{0}\alpha_{5}^{4}}{-625\beta_{1}^{4}\alpha_{4}\alpha_{3}^{4}+C\left(-\alpha_{4}^{5}+3125\alpha_{0}\alpha_{5}^{4}\right)\exp\left\{-4\frac{-\alpha_{4}^{5}+3125\alpha_{0}\alpha_{5}^{4}}{625\alpha_{4}\alpha_{5}^{3}}t\right\}}\right\}}^{\frac{1}{4}} \end{array}$$

The obtaining of exact solutions of the chain of equations can be continued. Below, we focus on the properties of the news waves described by the solutions (10) and (11). The solutions (12) and (13) are also possible solutions for the specific cases of the studied chain of equations. The application of these solutions to the situations modeled by the SIR model is limited because of the relative large number of relationships among the parameters $a_{i}$ (see Appendix B). Because of this, we will not discuss them below.

We note that we are interested in solutions for which I≥0, R≥0, and S≥0. This is because the numbers of susceptible, infected, and recovered individuals cannot be negative. In addition, we are not interested in the fixed points connected to the model and in stability of these fixed points. We are interested in solutions which can describe news waves.

## 3. Discussion of the Obtained Exact Analytical Solutions of the Studied Chain of Equations

Above, we have presented analytical relationships for the quantity R(t). This allows us to calculate the time evolution of the active in the posting (interesting in the piece of news) persons *I* on the basis of (2). Then, we can calculate the relative growth rate from (3) and $R_{n}\left(t\right)$ from (4). Our basic approximation for the reducing the SIR model to a chain of equations was $\frac{e}{(M+1)!}{\left(\frac{\tau R}{\rho N}\right)}^{M+1}<<1$. This means that the news wave has to affect a relatively small number of the entire population. If this is not the case, we have to solve the SIR model numerically.

We have analytical relationships for several news waves. Thus, we can calculate their characteristics by means of the relationships, mentioned above. For the calculation of *S* we use the approximate relationship, which occurs from (5):

(14) $$S\left(t\right)=S\left(0\right)\left[1-\frac{\tau R}{\rho N}\right]$$

We start from the specific solution (10). From the requirement R(0)=0, we obtain for the constant of integration *C*:

(15) $$\begin{array}{c} C=\frac{2}{\theta }\mathrm{atanh}\left[\frac{\theta (\alpha_{1}E-2\alpha_{2}D)}{2\alpha_{1}\alpha_{2}D-\theta^{2}E}\right]. \end{array}$$

The solution (10) becomes:

(16) $$\begin{array}{c} R\left(t\right)=-\frac{\alpha_{1}}{2\alpha_{2}}-\frac{\theta }{2\alpha_{2}}\tanh\left[\frac{\theta \left\{t+\frac{2}{\theta }\mathrm{atanh}\left[\frac{\theta (\alpha_{1}E-2\alpha_{2}D)}{2\alpha_{1}\alpha_{2}D-\theta^{2}E}\right]\right\}}{2}\right]+\\ \frac{D}{\cosh^{2}\left[\frac{\theta \left\{t+\frac{2}{\theta }\mathrm{atanh}\left[\frac{\theta (\alpha_{1}E-2\alpha_{2}D)}{2\alpha_{1}\alpha_{2}D-\theta^{2}E}\right]\right\}}{2}\right]\left\{E-\frac{2\alpha_{2}D}{\theta }\tanh\left[\frac{\theta \left\{t+\frac{2}{\theta }\mathrm{atanh}\left[\frac{\theta (\alpha_{1}E-2\alpha_{2}D)}{2\alpha_{1}\alpha_{2}D-\theta^{2}E}\right]\right\}}{2}\right]\right\}} \end{array}$$

where (16) allows us to calculate the other quantities connected to this solution as follows:

(17) $$\begin{array}{c} I=\frac{1}{\rho }\frac{dR}{dt}=\frac{1}{\rho }\{\frac{\theta^{2}}{4\alpha_{2}}\left\{1-\tanh^{2}\left[\frac{\theta \left\{t+\frac{2}{\theta }\mathrm{atanh}\left[\frac{\theta (\alpha_{1}E-2\alpha_{2}D)}{2\alpha_{1}\alpha_{2}D-\theta^{2}E}\right]\right\}}{2}\right]\right\}-\\ \frac{D\theta \tanh\left[\frac{\theta \left\{t+\frac{2}{\theta }\mathrm{atanh}\left[\frac{\theta (\alpha_{1}E-2\alpha_{2}D)}{2\alpha_{1}\alpha_{2}D-\theta^{2}E}\right]\right\}}{2}\right]\left\{1-\tanh^{2}\left[\frac{\theta \left\{t+\frac{2}{\theta }\mathrm{atanh}\left[\frac{\theta (\alpha_{1}E-2\alpha_{2}D)}{2\alpha_{1}\alpha_{2}D-\theta^{2}E}\right]\right\}}{2}\right]\right\}}{E-\frac{2\alpha_{2}D\tanh\left[\frac{\theta \left\{t+\frac{2}{\theta }\mathrm{atanh}\left[\frac{\theta (\alpha_{1}E-2\alpha_{2}D)}{2\alpha_{1}\alpha_{2}D-\theta^{2}E}\right]\right\}}{2}\right]}{\theta }}+\\ D^{2}\alpha_{2}\frac{{\left\{1-\tanh^{2}\left[\frac{\theta \left\{t+\frac{2}{\theta }\mathrm{atanh}\left[\frac{\theta (\alpha_{1}E-2\alpha_{2}D)}{2\alpha_{1}\alpha_{2}D-\theta^{2}E}\right]\right\}}{2}\right]\right\}}^{2}}{{\left\{E-\frac{2\alpha_{2}D}{\theta }\tanh\left[\frac{\theta \left\{t+\frac{2}{\theta }\mathrm{atanh}\left[\frac{\theta (\alpha_{1}E-2\alpha_{2}D)}{2\alpha_{1}\alpha_{2}D-\theta^{2}E}\right]\right\}}{2}\right]\right\}}^{2}}\} \end{array}$$

In addition:

(18) $$\begin{array}{c} S\left(t\right)=S\left(0\right)\{1-\frac{\tau }{\rho N}\{-\frac{\alpha_{1}}{2\alpha_{2}}-\frac{\theta }{2\alpha_{2}}\tanh\left[\frac{\theta \left\{t+\frac{2}{\theta }\mathrm{atanh}\left[\frac{\theta (\alpha_{1}E-2\alpha_{2}D)}{2\alpha_{1}\alpha_{2}D-\theta^{2}E}\right]\right\}}{2}\right]+\\ \frac{D}{\cosh^{2}\left[\frac{\theta \left\{t+\frac{2}{\theta }\mathrm{atanh}\left[\frac{\theta (\alpha_{1}E-2\alpha_{2}D)}{2\alpha_{1}\alpha_{2}D-\theta^{2}E}\right]\right\}}{2}\right]\left\{E-\frac{2\alpha_{2}D}{\theta }\tanh\left[\frac{\theta \left\{t+\frac{2}{\theta }\mathrm{atanh}\left[\frac{\theta (\alpha_{1}E-2\alpha_{2}D)}{2\alpha_{1}\alpha_{2}D-\theta^{2}E}\right]\right\}}{2}\right]\right\}}\}\} \end{array}$$

where σ(t) and $R_{n}\left(t\right)$ can be calculated from (3) and (4).

The above results are valid if $\frac{e}{3!}{\left(\frac{\tau R}{\rho N}\right)}^{3}\approx {\left(\frac{\tau R}{\rho N}\right)}^{3}<<1$. This means that:

(19) $$\begin{array}{c} \frac{\tau^{2}}{\rho^{2}N^{2}}\{-\frac{\alpha_{1}}{2\alpha_{2}}-\frac{\theta }{2\alpha_{2}}\tanh\left[\frac{\theta \left\{t+\frac{2}{\theta }\mathrm{atanh}\left[\frac{\theta (\alpha_{1}E-2\alpha_{2}D)}{2\alpha_{1}\alpha_{2}D-\theta^{2}E}\right]\right\}}{2}\right]+\\ \frac{D}{\cosh^{2}\left[\frac{\theta \left\{t+\frac{2}{\theta }\mathrm{atanh}\left[\frac{\theta (\alpha_{1}E-2\alpha_{2}D)}{2\alpha_{1}\alpha_{2}D-\theta^{2}E}\right]\right\}}{2}\right]\left\{E-\frac{2\alpha_{2}D}{\theta }\tanh\left[\frac{\theta \left\{t+\frac{2}{\theta }\mathrm{atanh}\left[\frac{\theta (\alpha_{1}E-2\alpha_{2}D)}{2\alpha_{1}\alpha_{2}D-\theta^{2}E}\right]\right\}}{2}\right]\right\}}{\}}^{3}<<1 \end{array}$$

For t=0, R(0)=0 and ${\left(\frac{\tau R}{\rho N}\right)}^{3}<<1$ is satisfied. For large values of *t*, $R\approx -\frac{\alpha_{1}}{2\alpha_{2}}-\frac{\theta }{2\alpha_{2}}$, and ${\left(\frac{\tau R}{\rho N}\right)}^{3}<<1$ can be satisfied.

Next, we discuss the solution (11). In this case, we have one relationship among $\alpha_{0}$, $\alpha_{1}$, $\alpha_{2}$, and $\alpha_{3}$. This leads to the following relationship for ρ:

(20) $$\rho =\tau \left[1-\frac{S(0)}{3N}\right]$$

For the constant of integration *C* we obtain:

(21) $$C=54\beta_{1}^{2}\frac{N-S(0)}{54N^{3}-5S{\left(0\right)}^{3}+36NS{\left(0\right)}^{2}-81S\left(0\right)N^{2}}$$

This leads to the following result for R(t):

(22) $$\begin{array}{c} R\left(t\right)=\left[N-\frac{S(0)}{3}\right]\left\{1-{\left[\frac{6N-5S(0)}{S\left(0\right)+6\left[N-S\left(0\right)\right]\exp\left(\frac{[6N-5S(0)]\tau t}{3N}\right)}\right]}^{1/2}\right\}. \end{array}$$

Then:

(23) $$I\left(t\right)=\frac{\left[N-S\left(0\right)\right]{\left[6N-5S\left(0\right)\right]}^{3/2}\exp\left(\frac{[6N-5S(0)]\tau t}{3N}\right)}{{\left[S\left(0\right)+6\left[N-S\left(0\right)\right]\exp\left(\frac{[6N-5S(0)]\tau t}{3N}\right)\right]}^{3/2}}$$

(24) $$S\approx S\left(0\right)\left\{1-\frac{\tau }{\rho N}\left[N-\frac{S(0)}{3}\right]\left\{1-{\left[\frac{6N-5S(0)}{S\left(0\right)+6\left[N-S\left(0\right)\right]\exp\left(\frac{[6N-5S(0)]\tau t}{3N}\right)}\right]}^{1/2}\right\}\right\}$$

On the bass of the obtained relationships, we can easily calculate σ(t) and $R_{n}\left(t\right)$ by use of (3) and (4).

Next, we have to check if the solution (22) satisfies the condition $\frac{e}{4!}{\left(\frac{\tau R}{\rho N}\right)}^{5}<<1$. At t=0, the condition is satisfied as R(0)=0. For large values of *t*, we have $R\approx \left[N-\frac{S(0)}{3}\right]$. The condition is equal to $\frac{e}{4!}$, which is about 0.113 and we can assume that this is small enough in comparison to 1.

## 4. Characteristics of the News Waves Based on the Solutions (16) and (11)

We proceed as follows. First, we discuss the case D=0. Then, we discuss the corrections connected to the case D≠0.

### 4.1. D=0

In this case, (16) becomes:

(25) $$\begin{array}{c} R\left(t\right)=-\frac{\alpha_{1}}{2\alpha_{2}}-\frac{\theta }{2\alpha_{2}}\tanh\left\{\frac{\theta }{2}[t+\frac{2}{\theta }\mathrm{atanh}(-\frac{\alpha_{1}}{\theta }\right)]\} \end{array}$$

In order to use this solution for the study of news waves we have to substitute the relationships for the coefficients $\alpha_{i}$ and θ in it. For θ, we obtain:

(26) $$\theta ={\left[{\left(\frac{\tau S(0)}{N}-\rho \right)}^{2}+2\frac{\tau^{2}S\left(0\right)\left[N-S\left(0\right)\right]}{N^{2}}\right]}^{1/2}.$$

The result for R(t) is:

(27) $$\begin{array}{c} R\left(t\right)=\frac{\rho N^{2}}{\tau^{2}S\left(0\right)}\{\frac{\tau S(0)}{N}-\rho +{\left[{\left(\frac{\tau S(0)}{N}-\rho \right)}^{2}+2\frac{\tau^{2}S\left(0\right)\left[N-S\left(0\right)\right]}{N^{2}}\right]}^{1/2}\tanh\{t+\\ \frac{2}{{\left[{\left(\frac{\tau S(0)}{N}-\rho \right)}^{2}+2\frac{\tau^{2}S\left(0\right)\left[N-S\left(0\right)\right]}{N^{2}}\right]}^{1/2}}\mathrm{atanh}\left\{\frac{\rho -\frac{\tau S(0)}{N}}{{\left[{\left(\frac{\tau S(0)}{N}-\rho \right)}^{2}+2\frac{\tau^{2}S\left(0\right)\left[N-S\left(0\right)\right]}{N^{2}}\right]}^{1/2}}\right\}\}\} \end{array}$$

Next, *I* can be obtained by substitution of $\alpha_{0}$, $\alpha_{1}$, $\alpha_{2}$ and θ in (17). Furthermore, finally, we have:

(28) $$\begin{array}{c} S\approx S\left(0\right)\{1-\frac{N}{\tau S(0)}\{\frac{\tau S(0)}{N}-\rho +{\left[{\left(\frac{\tau S(0)}{N}-\rho \right)}^{2}+2\frac{\tau^{2}S\left(0\right)\left[N-S\left(0\right)\right]}{N^{2}}\right]}^{1/2}\tanh\{t+\\ \frac{2}{{\left[{\left(\frac{\tau S(0)}{N}-\rho \right)}^{2}+2\frac{\tau^{2}S\left(0\right)\left[N-S\left(0\right)\right]}{N^{2}}\right]}^{1/2}}\mathrm{atanh}\left\{\frac{\rho -\frac{\tau S(0)}{N}}{{\left[{\left(\frac{\tau S(0)}{N}-\rho \right)}^{2}+2\frac{\tau^{2}S\left(0\right)\left[N-S\left(0\right)\right]}{N^{2}}\right]}^{1/2}}\right\}\}\}\} \end{array}$$

**Figure 1:**
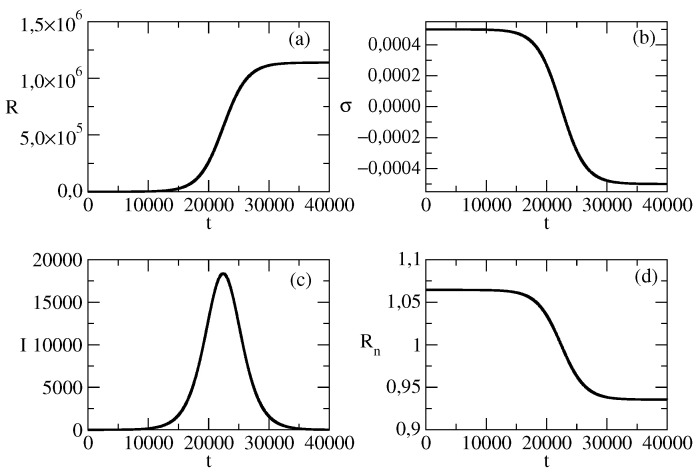
The basic solution for all figures below.$N=10^{7}$, S(0) = 9,999,999, τ=0.00825, ρ=0.00775. (**a**): R(t). (**b**): σ(t). (**c**): I(t). (**d**): $R_{n}\left(t\right)$.

Figure 1 shows the basic solution for all of the following figures, where we will show the influence of the changes of parameters on this basic solution. Figure 1a shows the number of individuals affected by the news wave. The number of individuals who are active in posting the piece of news is shown in Figure 1c. Figure 1b shows the growth rate σ for the basic solution. Figure 1d shows the effective reproduction rate for the spread of the wave connected to the piece of the news.

**Figure 2:**
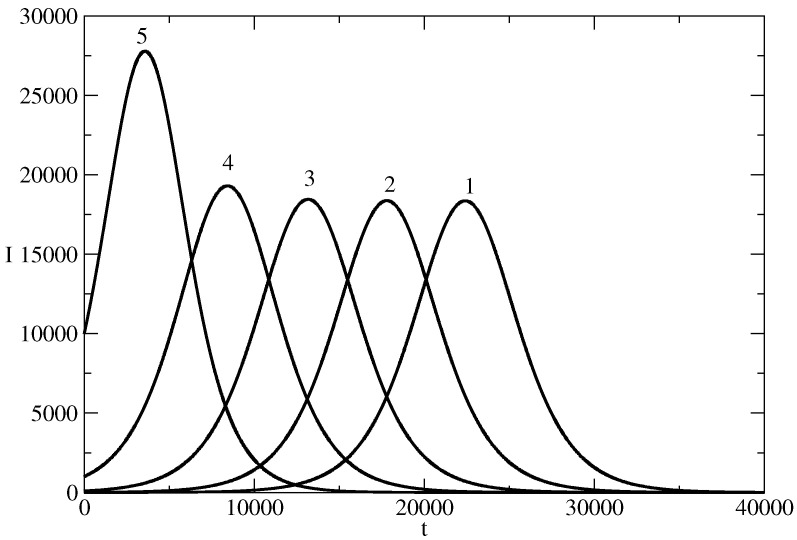
Influence of S(0) on the news wave. The profile of *I* for the basic solution $N=10^{7}$, S(0) = 9,999,999, τ=0.00825, ρ=0.00775 is denoted by 1. In other curves, we changed only the values of S(0). Curve 2: S(0) = 9,999,990. Curve 3: S(0) = 9,999,900. Curve 4: S(0)= 9,999,000. Curve 5: S(0) = 9,990,000.

Figure 2 shows the influence of the parameter S(0) on the news wave. As R(0)=0, then I(0)=N−S(0), and in fact, the figure shows the influence of the initial number of individuals who start to spread the piece of news on the evolution of the number of individuals who spread the piece of news at the time of the existence of the news wave. Curve 1 is for the case of the basic solution, which is for I(0)=1 individual who starts to spread the piece of the news. Curve 2 is for I(0)=10 individuals who start to spread the piece of news. We see that with an increase of I(0), there is a tendency to increase in the amplitude of the news wave and the maximum of the wave occurs earlier (the time horizon of the wave is shorter). This means that by manipulation of I(0) one can have a news wave of a certain amplitude at a selected moment in time.

**Figure 3:**
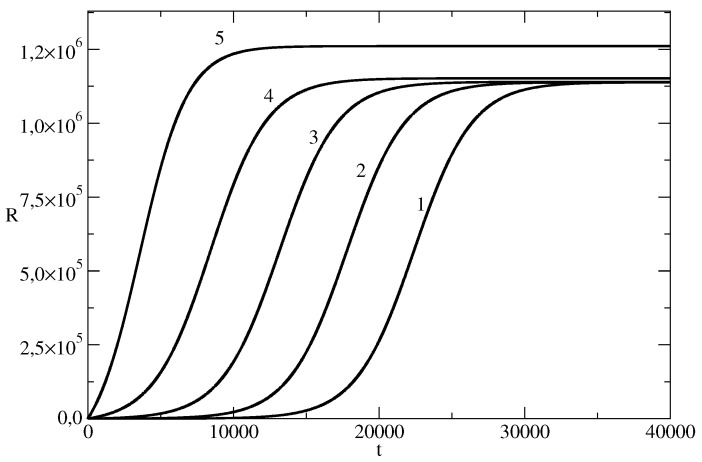
Influence of S(0) on the number *R* of the individuals affected by the news wave. *R* for the basic solution $N=10^{7}$, S(0) = 9,999,999, τ=0.00825, ρ=0.00775 is denoted by 1. In other curves, we changed only the values of S(0). Curve 2: S(0) = 9,999,990. Curve 3: S(0) = 9,999,900. Curve 4: S(0) = 9,999,000. Curve 5: S(0)= 9,990,000.

The influence of the initial number of individuals which start to spread the piece of news on the number of individuals affected by the news wave is shown in Figure 3. It can be seen that the number of affected individuals increases by decreasing S(0) (i.e., with an increasing I(0)).

**Figure 4:**
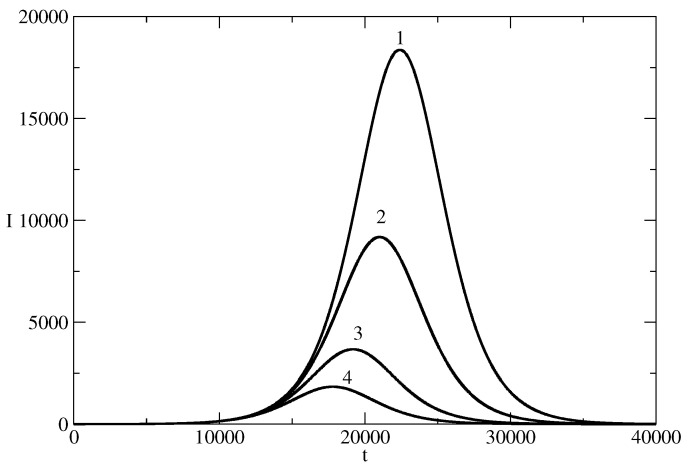
Influence of the population *N* on the news wave when S(0)=N−1 (i.e., I(0)=1) on the number *I* of the individuals who spread the piece of the news. *I* for the the basic solution $N=10^{7}$, S(0) = 9,999,999, τ=0.00825, ρ=0.00775 is denoted by 1. In other curves, we changed only the values of *N* and S(0)=N−1. Curve 2: N= 5,000,000. Curve 3: N= 2,500,000. Curve 4: N= 1,000,000.

Figure 4 shows the influence of the population *N* on the size of the news wave in the case when I(0)=1. In other words, when a single individual starts to spread the piece of news at the beginning of the wave. We observe two effects. First of all, a larger population leads to a larger wave amplitude. The second effect is quite interesting: a larger population leads to a slower wave and the peak of the wave moves to a larger time (the time horizon of the wave is larger). This means that for the same wave parameters, in countries with a larger population, the news waves peak is larger and the news wave exists for a longer time in comparison to the corresponding news wave in a country with a smaller population.

**Figure 5:**
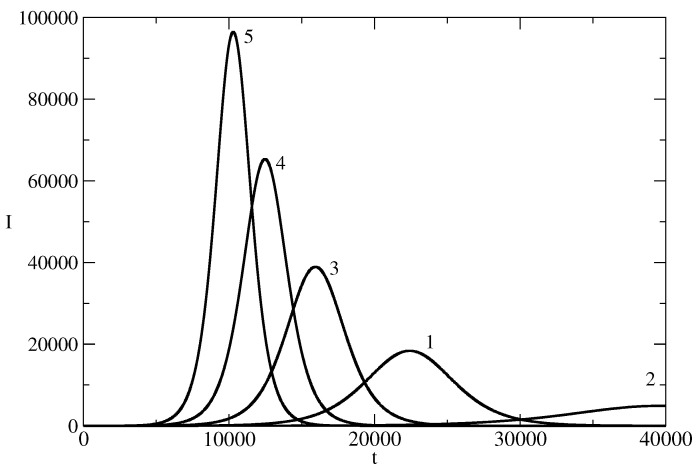
Influence of the transmission rate τ on the news wave. *I* for the the basic solution $N=10^{7}$, S(0)= 9,999, 999, τ=0.00825, ρ=0.00775 is denoted by 1. In other curves, we changed only the values of τ. Curve 2: τ=0.008. Curve 3: τ=0.0085. Curve 4: τ=0.0875. Curve 5: τ=0.009.

Figure 5 shows the influence of the transmission rate τ on the news wave. A larger transmission rate means that the piece of news spread easily in the corresponding population. The basic solution is marked by 1 on the figure. We see that the decrease in the value of the transmission rate leads to a news wave of smaller amplitude. In addition, the news wave develops slowly in time. The increase of the transmission rate leads to news waves of a larger amplitude. The time horizon of the wave becomes smaller.

**Figure 6:**
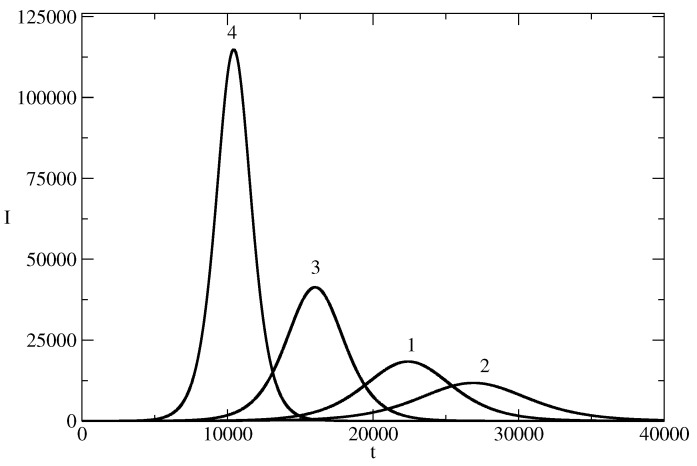
Influence of the recovery rate ρ on the news wave. *I* for the the basic solution $N=10^{7}$, S(0) = 9,999,999, τ=0.00825, ρ=0.00775 is denoted by 1. In other curves, we changed only the values of S(0) (I(0)). Curve 2: ρ=0.00785. Curve 3: ρ=0.075. Curve 4: ρ=0.7.

Figure 6 shows the influence of the changes of the value of the recovery rate ρ on the news wave. The basic wave is marked by 1. We see that the increase in the recovery rate leads to smaller amplitude of the news wave. The decrease of the value of the recovery rate leads to an increase in the wave amplitude and a smaller time horizon $t_{m}$ of the wave.

**Figure 7:**
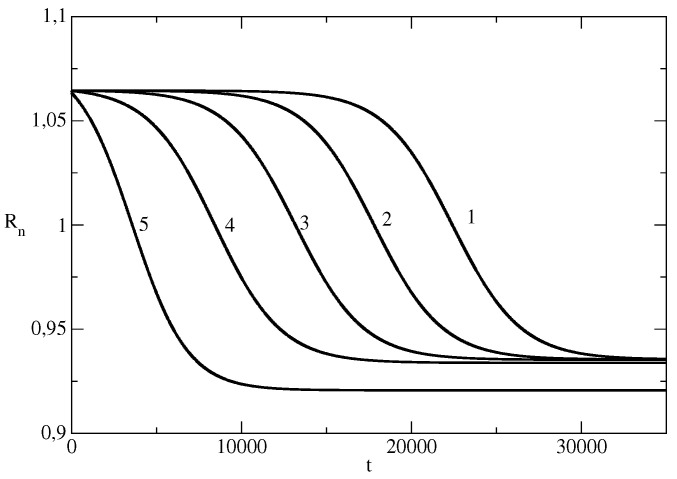
Effective reproduction number $R_{n}$ for the news wave for different values of $R_{n}$. *I* for the basic solution $N=10^{7}$, S(0) = 9,999,999, τ=0.00825, ρ=0.00775 is denoted by 1. In other curves, we changed only the values of S(0) (I(0)). Curve 2: I(0)=10. Curve 3: I(0)=100. Curve 4: I(0)=1000. Curve 5: I(0) = 10,000.

Figure 7 shows how the changes in the initial number I(0) of individuals which spread the piece of news affects the effective reproduction number of the news wave. A smaller value of I(0) leads to maintaining a larger $R_{n}$ for a longer time.

**Figure 8:**
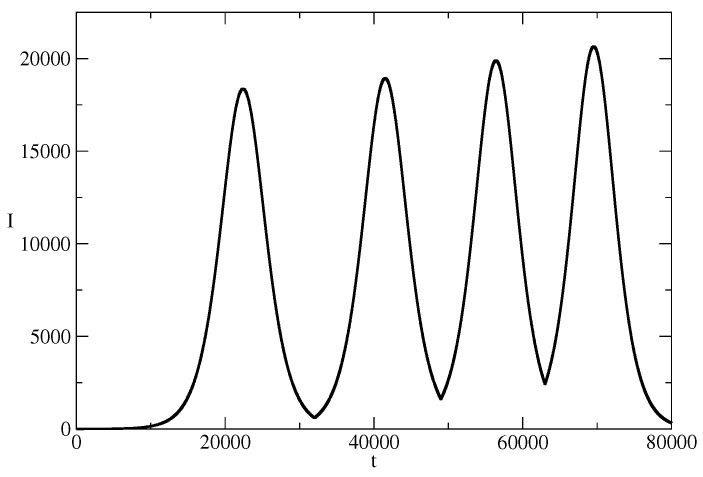
A news wavetrain. The train is made by four waves, having the parameters of the basic solution from Figure 1. The wavetrain is an illustration of the possibility for long presence in information space with increasing influence on the people in the same time.

Figure 8 illustrates the concept of the news wavetrain. The news wavetrain is a possible technology for increasing influence by a sequence of news. Let us have a piece of news and this piece of news starts to spread in a population. I(t) is the number of individuals who spread the news at the time *t*. At the time $t_{1}$ and before the vanishing of the first wave of the train, we have $I(t_{1})$ spreaders of the corresponding news. One can use these active spreaders in order to start the second wave of the wavetrain at $t=t_{1}$. The second wave of the train can contain a piece of news which is similar but slightly different from the piece of news spread by the first wave. Figure 8 illustrates the case when the initial number of spreaders of the second piece of news $I(t_{1})$ is larger than the initial number I(0) of spreaders of the first piece of news. This can lead to increase of the amplitude of the second wave of the news wavetrain with respect of the amplitude of the first wave of the train. In the same manner, one can start the third wave of the train at $t=t_{2}$ and for $I\left(t_{2}\right)>I\left(t_{1}\right)$. This can lead to even larger amplitude of the third wave of the news wavetrain. The process can be continued (Figure 8 shows a wavetrain containing four news waves). In such a way, one can achieve two effects simultaneously.

We will call the news wavetrain from Figure 8 an increasing news wavetrain because of the increasing amplitude of the news waves of the train. Construction of decreasing wavetrain is also possible. The third possibility is construction of a mixed amplitude news wavetrain where the amplitude of the waves of the train increases or decreases. Such wavetrain can have, for example, a similar shape as the wavetrains known from physics.

We note that an increasing news wavetrain can be constructed of news waves which have different values of the parameters ρ, θ, and $I(t_{n})$ at the beginning of the n−1-st wave of the train. The increasing news wavetrain is a phenomenon which can be useful in the case of the spreading of systems of ideas, in advertising, in propaganda, etc.

1. One is present for a long time in the information part of the mind of the population and a possibility for significant influence of this mind occurs.
2. At the same time, one can affect a larger and larger part of this population.

### 4.2. D≠0

In this case, we have to substitute $\alpha_{0}$, $\alpha_{1}$, $\alpha_{2}$, and θ from (8) and (26) in (16)–(18). As the corresponding relationships become quite long, we will not write them here. Instead of this, we will evaluate the correction in the case D≠0 in comparison to the case D=0. In order to avoid the large calculations, we consider the specific case t=−C. In this case, from (10), we obtain:

(29) $$R\left(-C\right)=-\frac{\alpha_{1}}{2\alpha_{2}}+\frac{D}{E}$$

where *C* is given by (15) and we have C<0 as the time *t* has to be positive. θ is larger than 0 and then we have that: $-\frac{1}{\theta }<\frac{\alpha_{1}E-2\alpha_{2}D}{2\alpha_{1}\alpha_{2}D-\theta^{2}E}<0$. Let us assume further that D>0, E>0, and $\alpha_{1}>0$. $\alpha_{2}<0$ by definition. Thus, $2\alpha_{1}\alpha_{2}D-\theta^{2}E<0$ and $\alpha_{1}E-2\alpha_{2}D>0$. Then, C<0 as required. From $-\frac{1}{\theta }<\frac{\alpha_{1}E-2\alpha_{2}D}{2\alpha_{1}\alpha_{2}D-\theta^{2}E}$, we obtain $\frac{D}{E}<\frac{\theta }{-2\alpha_{2}}$. The upper bound of D/E at t=−C is $\frac{\theta }{-2\alpha_{2}}$ and then the upper bound of R(−C) is:

(30) $$R\left(-C\right){|}_{upper\;bound\;D\neq 0}=-\frac{\alpha_{1}}{2\alpha_{2}}-\frac{\theta }{2\alpha_{2}}$$

We note that θ>0 and $\alpha_{2}<0$. At the same value of t=−C we have from (25):

(31) $$\begin{array}{c} R\left(-C\right){|}_{D=0}=-\frac{\alpha_{1}}{2\alpha_{2}} \end{array}$$

### 4.3. The Solution (11)

The quantities connected to this solution are given by (20)–(24). The solution describes a specific kind of news wave: we have a news wave which decreases with increasing time and the amplitude of the wave depends very much on the initial numbers of the spreaders of the piece of news.

**Figure 9:**
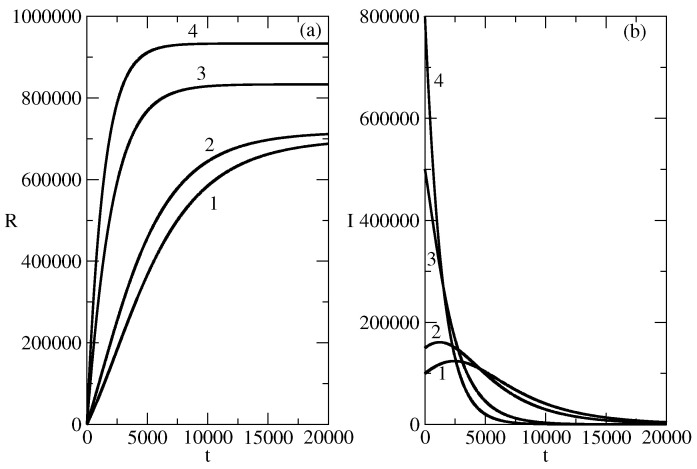
The solution (11). (**a**): Number of individuals affected by the news wave. Curve 1: Basic solution. N= 1,000,000, S(0)= 900,000, τ=0.009. Curves 2–4 show the influence of the change of the initial number of spreaders I(0)=N−S(0) of the piece of news on the number of individuals affected by the news wave. For the basic solution $I\left(0\right)=10^{5}$ Curve 2: $I\left(0\right)=1.5\cdot 10^{5}$. Curve 3:$I\left(0\right)=5\cdot 10^{5}$. Curve 4: $I\left(0\right)=8\cdot 10^{5}$. (**b**): Influence of the number I(0) of initial spreaders of the piece of news on the profile of the news wave. Curve 1: Basic solution $I\left(0\right)=10^{5}$. Curve 2: $I\left(0\right)=1.5\cdot 10^{5}$, Curve 3: $I\left(0\right)=5\cdot 10^{5}$. Curve 4: $I\left(0\right)=8\cdot 10^{5}$.

Figure 9 shows the influence of the initial number I(0) of the individuals spreading the piece of news on the shape and the time horizon of the news wave of the kind (11). In order to have a large wave of this kind, one has to start with a large number I(0). For example, in a population of $10^{6}$ individuals on has to start by $10^{5}$ initial spreaders of the piece of news in order to reach about $7\times 10^{5}$ individuals (Curve 1 of Figure 9a). Figure 9b shows that the increase of the number of initial spreaders decreases the time horizon of the wave and for large number of I(0) the time horizon of the wave becomes 0. Thus, the waves of the kind (11) require the presence of a large organization (in order to mobilize a large number of initial spreaders) and the waves are quite “dissipative” (the number of spreaders decrease very fast).

**Figure 10:**
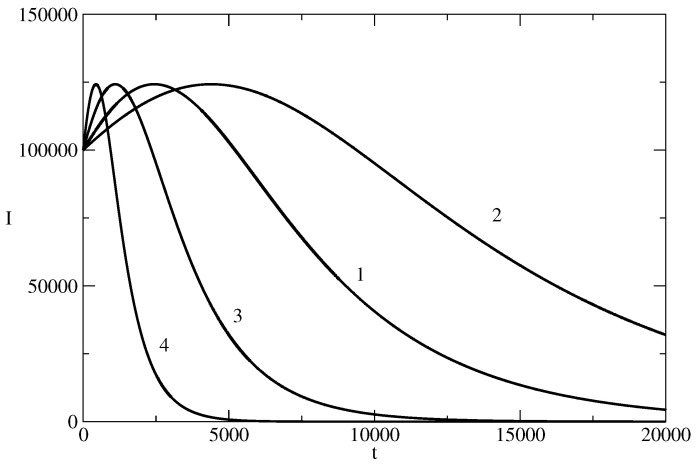
The solution (11). Influence of the transmission rate τ on the number of spreaders of the piece of news. Curve 1: Basic solution. N= 1,000,000, S(0)= 900,000, τ=0.009. Curve 2: τ=0.0005. Curve 3: τ=0.002. Curve 4: τ=0.005.

Figure 10 shows the influence of the change of the transition rate τ on the shape and on the time horizon of the wave. The increase of the value of the transition rate leads to a decrease of the time horizon of the wave and to the faster vanishing of the wave.

## 5. Discussion

The discussed theory leads to several hints about the possibility for manipulation of the size, amplitude, and time horizon of the news waves. They are as follows:

1. The organization of the process of initiation of a news wave is important because the amplitude and the time horizon of the news wave depend on the initial number of individuals which start to spread the corresponding piece of news. If one wants to have a news wave which possess a larger peak coming early in the time after the beginning of the wave, then, one has to organize a larger number I(0) of individuals which start to spread the piece of news. If one wants a larger time horizon, then I(0) must be smaller. However, this will lead to a news wave of smaller amplitude, i.e., the number of individuals affected by the news wave will be smaller.
2. Let us consider two cases of a region or a country. In the second case, the region or the country has a larger population in comparison to the first case. However, in both cases, the values of the parameters τ and ρ remain the same. In a region or country with a larger population, the amplitude of the news wave will be larger and the time horizon will be longer in comparison to a region or country of a smaller population. Thus, the time of “life” of a piece of news in a large (with respect to population) city or region or country is expected to be longer than the time of “life” of the same piece of news in a town, region, or country of smaller population (but having the same values of ρ and θ).
3. The transmission rate τ strongly influences the amplitude and the time horizon of the new wave. Thus, in order to achieve a news wave of larger amplitude (more affected individuals by the piece of news), one has to ensure a larger transmission rate (the corresponding population must be made more susceptible to the corresponding kind of news). However, the larger transmission rate also leads to a shorter time horizon. In other words, the news wave of larger amplitude moves faster through the population because of the higher permeability due to the larger transmission rate.
4. The increase of the recovery rate leads to a wave of smaller amplitude and larger time horizon. Thus, if one wants to achieve a news wave of larger amplitude, the recovery rate must be lowered. The appropriate selection of the recovery rate can fix the position of the peak of the news wave.
5. One can construct wave trains of news waves by using pieces of news with similar content. In such a case, one can use the number of individuals who spread the piece of news at a given time as the population of news spreaders who start to spread the next and slightly different piece of news. The wavetrains can be of three kinds. The news wavetrain which could be of interest to advertising or propaganda is the increasing news wavetrain which allows to affect the population of individuals whose number increases in time.

We can consider five types of news waves of the kind (25). Our classification will not be based on characteristics such as hardness of softness of the news or if the news is true or fake. The classification will be quantitative and it will be based on the parameters τ and ρ. We denote the types of news waves as: A-type, B-type, C-type, D-type, and E-type of news waves.

The A-type of news waves has a large value of the transmission rate and a small value of the recovery rate. This means that the population favors easy spread of the wave and the number of spreaders of the corresponding piece of news tends to decrease slowly. Such waves have a large amplitude and relatively small time horizon. The time horizon is small as the large amplitude compensates for the slow decrease of the number of spreaders as $\frac{dR}{dt}=\rho I$. Thus, the A-type of news wave affects a large number of individuals but lasts for a relatively short time period.

The B-type of news waves has a large value of the transmission rate and a large value for the recovery rate. The large value of τ favors a large amplitude and a small time horizon and the large value of ρ favors a smaller amplitude of the wave and a larger time horizon. Thus, the actual value of the wave amplitude and the time horizon of the B-type of news wave depend on the value of the ratio τ/ρ.

The C-type of news waves has a small value of the transmission rate and a large value of the recovery rate. The small value of τ favors a small amplitude and a large time horizon of the news wave. The small amplitude and the large time horizon are also favored by the large value of ρ. Thus, the C-type of news wave will have a typically small amplitude and a large time horizon.

The D-type of news waves has a small value of the transmission rate and a small value of the recovery rate. The small transmission rate means that the population is not very permeable by the news wave. This can be compensated by the small recovery rate, which means that the piece of the news affects the individuals of the population for a longer time. Thus, we can construct a wave possessing various values of the amplitude and time horizon, and this depends again on the ratio τ/ρ as in the case of the B-type wave, which, however, is connected to larger permeability and faster recovery in comparison to the D-type of news wave.

**Figure 11:**
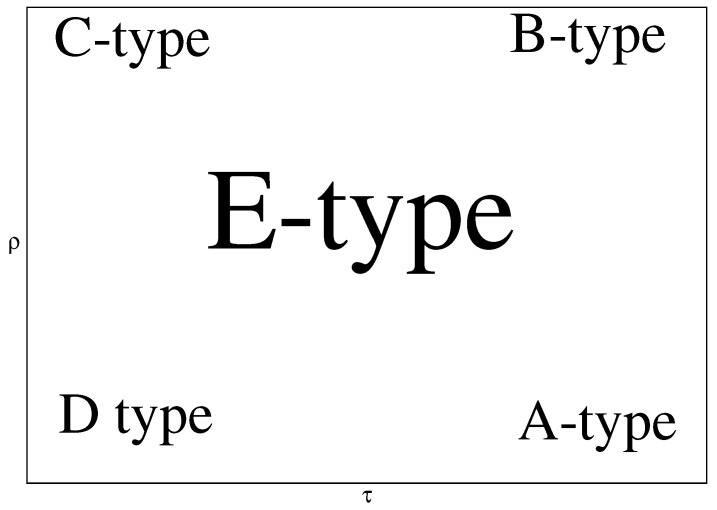
Types of news waves of the kind (25) in the τ–ρ-plane. A-type wave: large value of τ, small value of ρ. B-type wave: large value of τ large value of ρ. C-type wave: small value of τ, large value of ρ. D-type wave: small value of τ, small value of ρ. Type E-wave: values of τ and ρ which can not be associated with one of the remaining four types of waves.

Finally, the E-type of news wave is connected to the values of transmission rate and recovery rate, which are intermediate to the four other types of waves. By appropriate increases and decreases of the values of τ and ρ, the E-type of news wave can be transformed to different type of wave. For example, the increase of the values of τ and ρ transforms the E-type of wave to B-type of wave. The increase of τ and decrease of ρ transforms the E-type of wave to A-type of news wave (see Figure 11).

News waves are also possible on the basis of other solutions of equations from the studied chain of equations. Such waves require a prescribed relationship for the ratio between the transmission rate and the recovery rate. The increase of the transition rate for this wave leads to a shorter time horizon and to a faster decrease of the number of spreaders of the corresponding piece of news.

## 6. Concluding Remarks

In this article, we study the spread of news in a population. The basis of the study is the SIR model of the spread of epidemics. The model is reduced to a single equation which is associated with a chain of nonlinear differential equations which possess polynomial nonlinearities. By means of the Simple Equations Method (SEsM), we obtain exact solutions to several equations of this chain. We study the influence of the parameters of the model on the shape, peak, and the time horizon associated with the news waves. The presence of analytical solutions allows us to make many interesting conclusions about the possibility for change of the time horizon and maximum value of the wave. These conclusions give interesting hints for the practitioners.

The presented theory can be applied to various kinds of news, such as fake news, rumors, and for news in print media and news in social media. One can spread hard news or soft news and the corresponding news wave can have parameters τ and ρ, with different values depending on the regions. The difference in the values of the parameters of the news wave leads to different amplitude and different time horizon of the wave. One can try to manipulate these parameters in order to obtain, for example, large amplitude short time horizon hard news wave or small amplitude long time horizon soft news wave. In addition, we consider the possibility for construction of wavetrains from several news waves. Three kinds of wavetrains are possible. The most interesting for practice (in areas such as advertising, propaganda, etc.) is the increasing wavetrain. This wavetrain allows the presence of a source of news for a long time in the minds of a population, and at the same time, the number of individuals influenced by the news increases.

Finally, we note that this article was devoted to new waves for which the ratio R/N is small. This allow us to obtain analytical results and these analytical results are the focus of the article. In a future article, we plan to study the case when the ratio R/N has larger value and especially the case when R/N becomes close to 1. Such waves will be studied numerically. 

## Data Availability

No new data were created or analyzed in this study. Data sharing is not applicable to this article.

## Appendix A. Several Remarks about the Simple Equations Method (SEsM)

The Simple Equations Method (SEsM) was developed as a tool for the the modeling of various complex systems, such as atomic chains and lattices and large biological systems of animals or groups of humans, such as, for example, research groups or economic systems [62,63,64,65,66,67]. As the complex systems are nonlinear [68,69,70], they must be studied by the methods of nonlinear time series analysis and the models used for such systems contain very often nonlinear differential or difference equations [71,72,73,74,75,76,77,78,79,80]. The analytical solutions of these nonlinear equations are of large interest. Because of this, many researchers invest their efforts to obtain such solutions. We mention the Inverse Scattering Transform method [81] and the Method of Hirota [82], as well as the Method of Simplest equation of Kudryashov [83,84]. The methodology of SEsM [85,86,87,88,89,90] will be used in this text. The first steps in the development of SEsM have been made many years ago [91]. The work on the methodology continued in [92] and it was applied to problems of ecology. Further development of the methodology was made in [93] and numerous applications followed [94]. Since 2018, we use the current version of the methodology (SEsM). SEsM can use more than one simple Equation [95]. For more discussion and applications of SEsM, see [85,86,87,88,89,90,91,92,93,94,95,96,97,98].

The general notation of SEsM is SEsM(n,m). This means that we have to solve *n* nonlinear differential equations by means of the known solutions of *m* simpler equations. The notation for the case when we solve a single nonlinear differential equation is SEsM(1,m). The most-used case up to now is SEsM(1,1), which is called the Modified Method of Simplest Equation: we solve one complicated nonlinear differential equation by means of the known solutions of one more simple differential equation.

The idea of SEsM is to reduce the solved system of nonlinear differential equations to a specific form. The solved system is as follows:

(A1) $$D_{i}\left[v_{i1}\left(x,\ldots ,t\right),\ldots ,v_{in}\left(x,\ldots ,t\right)\right]=0,\;\;\;i=1,2,\ldots ,n.$$

where $D_{i}\left[v_{i1}\left(x,\ldots ,t\right),\ldots ,v_{in}\left(x,\ldots ,t\right),\ldots \right]$ may depend on the functions $v_{i1}\left(x,\ldots ,t\right),\ldots ,$$v_{in}\left(x,\ldots ,t\right)$ and their derivatives. $V_{ik}$ also can depend on one or more spatial coordinates. The goal of SEsM is reduction of (A1) to the specific form:

(A2) $$\sum_{i=1}^{n}a_{ij}\left(\ldots \right)E_{ij}=0,j=1,2,\ldots ,p_{i}.$$

where $E_{ij}$ are functions of the independent spatial variables and of the time, $a_{ij}$ are relationships among the parameters of the solved equations, parameters of the solutions, and the parameters of the solutions of the more simple equations, and $p_{i}$ is a parameter which is characteristic for the *i*-th solved equation. Note that the relationships $a_{ij}$ contain only parameters, whereas the spatial and time coordinates are concentrated on the functions $E_{ij}$. If we manage to reduce the solved equations to the form (A1) and we set $a_{ij}=0$, we may obtain a nontrivial solution of the system of nonlinear differential equations.

In order to reduce (A1) to (A2), one can take four steps. The first step is to use transformations which can remove the nonlinearities in (A1) or can reduce these nonlinearities to more treatable kind of nonlinearities (such as polynomial nonlinearities). In the second step, one constructs the solutions of the solved equations as composite function of simpler equations. The kind of composite functions and the kind of simple equations used are determined in Step 3 in order to come to the relationships (A2). Finally, one sets:

(A3) $$a_{ij}\left(\ldots \right)=0$$

This leads to a system of nonlinear algebraic relationships among the parameters of the solved equations, the parameters of the composite functions, and the parameters of the solutions of the more simple equations. Any nontrivial solution of (A3) leads to a solution of the system (A1).

## Appendix B. Several Exact Solutions of the Chain of Equations

We will apply SEsM to the following equation:

(A4) $$\frac{dR}{dt}=\sum_{j=0}^{M}\alpha_{j}R^{j},$$

We start by using the equation of Bernoulli:

(A5) $$\frac{dy}{dt}=py+qy^{m},\;\;\;m=2,3,\ldots ,$$

as a simple equation. The transformation $y=u^{1/(1-m)}$ reduces (A5) to a linear differential equation. In such a way, we obtain the solution of the equation of Bernoulli as follows:

(A6) $$y\left(t\right)={\left\{\frac{p}{-q+Cp\exp[-(m-1)pt]}\right\}}^{\frac{1}{m-1}}.$$

where *C* is a constant of integration.

Then, we construct the composite function R(y) as:

(A7) $$R\left(y\right)=\sum_{l=0}^{L}\beta_{l}y^{l}.$$

where y(t) is the solution of the equation of Bernoulli (A6). At the following step of SEsM, we obtain the balance equation. (A5) and (A7) lead to the balance equation:

(A8) m=1+L(M−1).

Then, we obtain a specific solution of (A4):

(A9) $$R\left(t\right)=\sum_{l=0}^{L}\beta_{l}{\left\{\frac{p}{-q+Cp\exp\{-[L(M-1)]pt\}}\right\}}^{\frac{l}{L(M-1)}}.$$

T he parameters $\beta_{l}$, *p*, *q*, and *C* will be obtained by solving the system of algebraic equations at the last step of SEsM.

For the case M=2, we can obtain the general solution of (A4). In this case, (A4) becomes:

(A10) $$\frac{dR}{dt}=\alpha_{2}R^{2}+\alpha_{1}R+\alpha_{0}.$$

where (A10) is an equation of the Riccati kind. We know a specific solution of (A10) as follows:

(A11) $$R\left(t\right)=-\frac{\alpha_{1}}{2\alpha_{2}}-\frac{\theta }{2\alpha_{2}}\tanh\left[\frac{\theta (t+C)}{2}\right],$$

In (A11), $\theta^{2}=\alpha_{1}^{2}-4\alpha_{0}\alpha_{2}>0$ and *C* is a constant of integration. On the basis of this specific solution, we can obtain the general solution of (A10). This general solution is $R=-\frac{\alpha_{1}}{2\alpha_{2}}-\frac{\theta }{2\alpha_{2}}\tanh\left[\frac{\theta (t+C)}{2}\right]+\frac{D}{v}$, where *D* is a constant. In addition, v(t) is the solution of the linear differential equation:

(A12) $$\frac{dv}{dt}-\theta \tanh\left[\frac{\theta (t+C)}{2}\right]vs.=-\alpha_{2}D$$

This solution is:

(A13) $$v=\cosh^{2}\left[\frac{\theta (t+C}{2}\right]\left\{E-\frac{2\alpha_{2}D}{\theta }\tanh\left[\frac{\theta (t+C}{2}\right]\right\}.$$

where *E* is a constant of integration. Thus, we obtain (10) as the general solution of the Equation (A10).

Next, we obtain several solutions of the kind (A9). For M=2 we have the general solution (10) of the Equation (A10). Thus, we continue by M=3. We note that the specific case for this text is that we consider only solutions for which L=1. For these solutions, it follows from (A9) that:

(A14) $$\begin{array}{c} R\left(t\right)=\beta_{0}+\beta_{1}{\left\{\frac{p}{-q+Cp\exp\{-[(M-1)]pt\}}\right\}}^{\frac{1}{\left(M-1\right)}}. \end{array}$$

The equation we have to solve for M=3 is:

(A15) $$\frac{dR}{dx}=\alpha_{3}R^{3}+\alpha_{2}R^{2}+\alpha_{1}R+\alpha_{0}.$$

The balance equation is m=3. This fixes the form of the simple equation of Bernoulli for this case: $\frac{dy}{dx}=py+qy^{3}$. The substitution of the last relationships in (A15) leads to the following system of nonlinear algebraic relationships:

(A16) $$\begin{array}{ccc} q-\alpha_{3}\beta_{1}^{2} & = & 0,\\ 3\alpha_{3}\beta_{0}+\alpha_{2} & = & 0,\\ p-3\alpha_{3}\beta_{0}^{2}-\alpha_{1}-2\alpha_{2}\beta_{0} & = & 0,\\ -\alpha_{1}\beta_{0}-\alpha_{3}\beta_{0}^{3}-\alpha_{0}-\alpha_{2}\beta_{0}^{2} & = & 0. \end{array}$$

The solution of (A16) is:

(A17) $$\begin{array}{c} p=\frac{3\alpha_{1}\alpha_{3}-\alpha_{2}^{2}}{3\alpha_{3}};\;\;\;q=\alpha_{3}\beta_{1}^{2};\;\;\;\beta_{0}=-\frac{\alpha_{2}}{3\alpha_{3}};\;\;\;\alpha_{0}=\frac{\alpha_{2}\left(9\alpha_{1}\alpha_{3}-2\alpha_{2}^{2}\right)}{27\alpha_{3}^{2}}. \end{array}$$

Thus, the following equation:

(A18) $$\frac{dR}{dx}=\alpha_{3}R^{3}+\alpha_{2}R^{2}+\alpha_{1}R+\frac{\alpha_{2}\left(9\alpha_{1}\alpha_{3}-2\alpha_{2}^{2}\right)}{27\alpha_{3}^{2}},$$

has the specific exact analytical solution (11). Note that we have one relationship among the parameters $a_{0}$, $a_{1}$, $a_{2}$, $a_{3}$ in (A17).

Next, we consider the case M=4. We have to solve the following equation:

(A19) $$\frac{dR}{dx}=\alpha_{4}R^{4}+\alpha_{3}R^{3}+\alpha_{2}R^{2}+\alpha_{1}R+\alpha_{0}.$$

The solution is of the kind (A14), and from (A8), we have the balance equation m=4. This fixes the form of the simple equation of Bernoulli for this case: $\frac{dy}{dx}=py+qy^{4}$. The substitution of the above relationships in (A19) leads to the following system of nonlinear algebraic relationships:

(A20) $$\begin{array}{ccc} q-\alpha_{4}\beta_{1}^{3} & = & 0,\\ \alpha_{3}+4\alpha_{4}\beta_{0} & = & 0,\\ 3\alpha_{3}\beta_{0}+\alpha_{2}+6\alpha_{4}\beta_{0}^{2} & = & 0,\\ p-3\alpha_{3}\beta_{0}^{2}-2\alpha_{2}\beta_{0}-4\alpha_{4}\beta_{0}^{3}-\alpha_{1} & = & 0,\\ -\alpha_{1}\beta_{0}-\alpha_{4}\beta_{0}^{4}-\alpha_{0}-\alpha_{3}\beta_{0}^{3}-\alpha_{2}\beta_{0}^{2} & = & 0. \end{array}$$

The solution of (A20) is:

(A21) $$\begin{array}{c} \beta_{0}=-\frac{\alpha_{3}}{4\alpha_{4}};\;\;\;q=\alpha_{4}\beta_{1}^{3};\;\;\;p=-\frac{\alpha_{3}^{4}-256\alpha_{0}\alpha_{4}^{3}}{64\alpha_{3}\alpha_{4}^{2}};\;\;\;\alpha_{1}=\frac{3\alpha_{3}^{4}+256\alpha_{0}\alpha_{4}^{3}}{64\alpha_{4}^{2}\alpha_{3}};\;\;\;\alpha_{2}=\frac{3\alpha_{3}^{2}}{8\alpha_{4}} \end{array}$$

Thus, the following equation:

(A22) $$\frac{dR}{dx}=\alpha_{4}R^{4}+\alpha_{3}R^{3}+\frac{\alpha_{3}^{2}}{8\alpha_{4}}R^{2}+\frac{3\alpha_{3}^{4}+256\alpha_{0}\alpha_{4}^{3}}{64\alpha_{4}^{2}\alpha_{3}}R+\alpha_{0},$$

has the solution (12). Note that we have two relationships among the parameters $a_{0}$, $a_{1}$,$a_{2}$, $a_{3}$, $a_{4}$ in (A21).

Next, we consider the case M=5. We have to solve the following equation:

(A23) $$\frac{dR}{dx}=\alpha_{5}R^{5}+\alpha_{4}R^{4}+\alpha_{3}R^{3}+\alpha_{2}R^{2}+\alpha_{1}R+\alpha_{0}.$$

The solution is of the kind (A7), and from (A8) we have the balance equation m=5. This fixes the form of the simple equation of Bernoulli for this case: $\frac{dy}{dx}=py+qy^{5}$. The substitution of the last relationships in (A23) leads to the following system of nonlinear algebraic relationships:

(A24) $$\begin{array}{ccc} q-\alpha_{5}\beta_{1}^{4} & = & 0,\\ \alpha_{4}+5\alpha_{5}\beta_{0} & = & 0,\\ \alpha_{3}+4\alpha_{4}\beta_{0}+10\alpha_{5}\beta_{0}^{2} & = & 0,\\ \alpha_{2}+3\alpha_{3}\beta_{0}+6\alpha_{4}\beta_{0}^{2}-10\alpha_{5}\beta_{0}^{3} & = & 0,\\ 3\alpha_{3}\beta_{0}^{2}+4\alpha_{4}\beta_{0}^{3}+5\alpha_{5}\beta_{0}^{4}+2\alpha_{2}\beta_{0}-p+\alpha_{1} & = & 0,\\ \alpha_{3}\beta_{0}^{3}+\alpha_{0}+\alpha_{5}\beta_{0}^{5}-\alpha_{1}\beta_{0}-\alpha_{2}\beta_{0}^{2}-\alpha_{4}\beta_{0}^{4} & = & 0. \end{array}$$

The solution of (A24) is as follows:

(A25) $$\begin{array}{c} q=\alpha_{5}\beta_{1}^{4};\;\;\;\;p=\frac{-\alpha_{4}^{5}+3125\alpha_{0}\alpha_{5}^{4}}{625\alpha_{4}\alpha_{5}^{3}};\;\;\;\beta_{0}=-\frac{\alpha_{4}}{5\alpha_{5}};\;\;\;\alpha_{1}=\frac{4\alpha_{4}^{5}+3125\alpha_{0}\alpha_{5}^{4}}{625\alpha_{5}^{3}\alpha_{4}};\\ \alpha_{2}=\frac{2\alpha_{4}^{3}}{25\alpha_{5}^{2}};\;\;\;\alpha_{3}=\frac{2\alpha_{4}^{2}}{5\alpha_{5}} \end{array}$$

Thus, the following equation:

(A26) $$\frac{dR}{dx}=\alpha_{5}R^{5}+\alpha_{4}R^{4}+\frac{2\alpha_{4}^{2}}{5\alpha_{5}}R^{3}+\frac{2\alpha_{4}^{3}}{25\alpha_{5}^{2}}R^{2}+\frac{4\alpha_{4}^{5}+3125\alpha_{0}\alpha_{5}^{4}}{625\alpha_{5}^{3}\alpha_{4}}R+\frac{\alpha_{4}^{5}}{3125\alpha_{5}^{4}},$$

has a specific solution (13). Note that we have two relationships among the parameters $a_{0}$, $a_{1}$, $a_{2}$, $a_{3}$, $a_{4}$, $a_{5}$ in (A25).

Obtaining the exact solutions of the chain of equations can be continued. We focus on the epidemic waves connected to the SIR model. The additional exact solutions of the chain of the equations will be discussed elsewhere.
